# Defining and understanding the relationship between professional identity and interprofessional responsibility: implications for educating health and social care students

**DOI:** 10.1007/s10459-017-9778-x

**Published:** 2017-05-17

**Authors:** Viktoria C. T. Joynes

**Affiliations:** Faculty of Health and Life Sciences, School of Medicine, Institute of Clinical Sciences, The University of Liverpool, Room 2.15, 2nd Floor, Cedar House, Ashton Street, Liverpool, L69 3GE UK

**Keywords:** Professional identity, Interprofessional education, Collaborative practice, Education, Interprofessional responsibility

## Abstract

This paper is concerned with exploring the relationship between perceptions of professional identities, interprofessional education (IPE) and collaborative practice. It seeks to introduce the concept of interprofessional responsibility as both a shift in the way in which to conceptualise the professional identity of Health and Social Care (H&SC) staff and as a new set of practices that help to inform the way in which students are prepared for collaborative working. The presented research, undertaken as part of a Ph.D. study, is based upon semi-structured interviews (n = 33) with H&SC staff who were recruited from both the United Kingdom (UK) Health Service and UK universities. Drawing upon thematic analysis of the data, the results of the research identified that previous conceptualisations of professional identity aligned to a whole profession do not relate to the way in which professionals perceive their identities. Senior professionals claimed to be more comfortable with their own professional identity, and with working across professional boundaries, than junior colleagues. Academic staff also identified that much IPE currently taught in universities serves the purpose of box-ticking rather than being delivered in meaningful way. It is proposed that the findings have implications for the way in which IPE is currently taught, and that adoption of the proposed concept of ‘interprofessional responsibility’ may help address some of the concerns these findings raise.

## Introduction

Individuals and professions are compelled to work in a collaborative way, for the benefit of patients, improved safety and in order to provide the highest possible standard of care. This compulsion is reinforced in the UK (where this study was undertaken) by policy introduced by the Department of Health and Quality Assurance Agency in 2006, that resulted in mandatory integration of interprofessional education (IPE) into pre-qualifying curricula by all regulatory bodies (Robson and Kitchen 2007; Craddock et al. 2013). Simultaneously, all health and social care (H&SC) professionals must still be trained to their own regulatory body’s professional standards, be able to perform their own roles, and carry out profession-specific tasks in line with their healthcare organisations’ requirements. Indeed, it has long been recognised that H&SC professionals must now work in an interprofessional way while maintaining their own discrete professional identity (Pirrie et al. 1999; Hornby and Atkins 2000). Simultaneously, the extent to which there is tension between perceptions of professional identities and collaborative practice has been a theme of IPE-related literature for many years (Nolan 1995; Hean et al. 2006; Pollard and Miers 2008).

Professional identity within health and social care professions is generally understood to develop at least in part through socialisation, a process explored in various ethnographic studies of different professions since the 1960s, many of which were influenced by Becker et al.’s (1961) longitudinal study ‘*Boys in White: student culture in medical school*’. Socialisation into a profession is a complex process, involving the impact of exposure to professional behaviour and interaction in the real world. As a result, placement experiences during pre-qualification training are commonly understood to be a key aspect of socialisation processes (Thompson and Ryan 1996). These processes have also been recognised as beginning to occur when people decide what they ‘want to be’ in their future career (‘anticipatory socialisation’—Flanagan 1979) and also as a continual process which carries on throughout the course of a career (Pratt et al. 2006; Cruess et al. 2015; Joseph et al. 2017).

The relationship between professional identity, IPE and collaborative practice remains complex. Professional identities have sometimes been depicted as a barrier to interprofessional education and working (Elston and Holloway 2001) with the suggestion that the struggle by each H&SC profession to define its own ‘sphere of practice and role in patient care’ is a major factor in determining the way in which the professions have developed in ‘silos’ (Hall 2005, p. 190). This has subsequently informed the way different professions have typically interacted. Emphasizing the multifaceted and intricate nature of the relationship between IPE and identities, Hean and Dickinson (2005) highlight that professional group membership has been variously argued to be something that needs to be emphasized (Hewstone et al. 2002) or alternatively underplayed (Gaertner et al. 1993) in order for IPE to change attitudes towards other professions successfully. Previously, IPE has also been portrayed as something with the potential to strengthen over time both individual professional identities and understandings of identities of others (Jakobsen et al. 2011). More recently, however, it has been suggested that introductory IPE courses such as those typical to most undergraduate education courses do not necessarily ‘strengthen’ professional identities, nor have a positive affect on changing attitudes towards other professions (Stull and Blue 2016).

Professional identity has typically been associated with the expectations that professions have of how professionals perform their roles, with the ‘internalising of professionalism’ being the ultimate aim of developing such an identity (Olckers et al. 2007, p. 2). In more recent years, it has been acknowledged that professional identity development is not something that H&SC students always understand or get ‘right’ without guidance, and recommendations have been made for more explicit educational objectives in curricula to cover not only professionalism, but also professional identity formation (Cruess et al. 2014, 2015) and cultural competencies such as what it means to be a ‘good doctor’ in different contexts (Sawatsky et al. 2017).

Despite this increase in focus on the way in which professional identities can and need to be developed, this does not appear to have yet had much impact on the way in which professional identities are conceptualised in practice. Certain responsibilities are still tied to certain professional identities, and conceptualisations of professional identity are commonly linked to single-professions, so that people performing those roles have such identities as, for example, ‘doctors’, ‘nurses’, ‘midwives’ or ‘social workers’. It is acknowledged here that this will be at least in part a symptom of the organisational frameworks under which H&SC operates; and that it is necessary both for the functioning of an intricate health care system, as well as politically, to group professionals under over-arching professional labels. Nevertheless there remains a paucity of evidence to suggest such groups exist as a single, cohesive community of practice that a shared professional identity label might imply. Indeed, it is this concentration on the development of a uni-professional identity that has been identified as causing difficulties for the introduction of IPE (Cameron 2011; Baker et al. 2011). As a result, attention has turned to the possibility of introducing a form of ‘interprofessional socialisation’ in which students are explicitly socialised into learning an interprofessional role and encouraged to develop a ‘dual identity’ incorporating a sense of belonging to both a professional group and an interprofessional community (Khalili et al. 2013; DiVall et al. 2014). Such socialisation becomes more urgent for newer roles such as physician’s assistants and clinical support workers, whose roles are designed to cross professional boundaries, but whose identities are less well defined, which may cause tension for such practitioners.

## Background to the study

This study emerged from a large-scale collaboration involving sixteen health and social care professions, that aimed to develop interprofessional working and assessment practices. During this collaboration, there was much discussion on the ‘tribalistic’ nature of H&SC professions and the way in which allegiances to professions were sometimes depicted as a barrier to people from different professions working successfully together (Carlisle et al. 2004). As an outsider to H&SC, it appeared that some of the opinions expressed by staff involved in the programme were indicative that *their* opinions could in fact be one of the main barriers to an interprofessional programme of work being implemented successfully. Resultantly, this study was designed to explore what IPE qualified staff had themselves experienced, and how this linked to their own conceptualizations of professional identity (if at all), in order to consider the impact of these experiences and their opinions of them on IPE programmes they were subsequently involved in facilitating.

The overarching aim of the study was to understand how qualified H&SC professionals perceive their own professional identities and how this relates to what they consider their professional roles and boundaries.

The following research questions guided the study:

1. How do H&SC staff conceptualise their professional identity, and the professional identity of other professions with whom they work or learn?
2. Do H&SC staff perceive that professional identities are reinforced, challenged or changed by interprofessional education and/or collaborative practice?
3. What implications do conceptualisations of professional identities and IPE have for the implementation of educational initiatives aimed at improving teamwork between professions for the ultimate aim of improving service user care?

## Methods

Data in this paper is drawn from a series of semi-structured interviews undertaken in England exploring the relationship between perspectives of H&SC staff on professional identity and experiences of IPE and collaborative practice, undertaken as part of a PhD study. The research was carried out with staff as opposed to students for two key reasons:

1. Given the influence of staff over socialisation processes, staff attitudes toward professional identity was viewed to potentially yield more knowledge about the successful implementation of initiatives which lead to more effective collaborative practice.
2. Staff hold a professional identity—that of a practicing professional. Students are likely to be still developing ‘professional identity[ies]’ and, at the least, would potentially have a dual identity associated with being a student of a profession.

Furthermore, staff were split into two categories, defined as ‘practicing’, that is, they were employed by the NHS, or ‘academic’, who were employed by universities; although it is acknowledged that the majority of practicing staff had some teaching role, and some of the academic staff (albeit a minority, all from medicine) were still also ‘practicing’ professionals. Participants are identified in the findings as practicing staff (coded as NHS-xx) or academic staff (coded as Higher Education Institution—HEI-xx). The decision to define staff in these two groups was again based upon the experience of working in a large-scale interprofessional programme. For IPE to have a lasting impact, it was apparent from an early stage that it was necessary for classroom-based initiatives to be backed up by placement learning experiences, and vice versa. For any impact to be achieved, both academic and practicing staff needed to be ‘on message’ as regards IPE and collaborative practice. Subsequently the study was designed to collect and look at the opinions of staff as defined by what they saw as their primary role (either as a practicing professional or an academic).

Participants were recruited for interview via two methods. Most were recruited via volunteering through completion of an earlier phase of the research, which was an online survey designed to collect opinions on IPE. All the practicing (NHS) participants and a third of the academic staff were recruited in this way. Due to low completion rate of the survey sent to academic staff (and subsequently not having enough potential interviewees) it was necessary to recruit further interviewees by email. Potential contacts were drawn from the five institutions who had been involved in the earlier IPE collaboration on which the study was based, who then passed the study details to colleagues.

The interview schedule was piloted with three participants, who then gave feedback on the questions. No substantial changes were made after piloting, although the order of the questions was changed slightly. The approach to the research was phenomenological, with a focus on encouraging respondents to describe what the most important aspects were for them of professional identity and IPE. The purpose of choosing such an approach was to allow the findings to emerge from the data; to allow for an exploratory study around the topics of professional identity and IPE where findings would be established through searching for ‘themes of meaning’ in responses (Rossman and Rallis 2003, p. 276), while ensuring the analysis remained grounded in the data as much as possible. Subsequently the interview schedule consisted of a list of open-ended questions (and follow-up prompts if they appeared relevant), to allow for exploration of key themes led by participant’s own priorities and perceptions. A copy of the full interview schedule is included in “Appendix”. Interview length was usually about an hour but did vary depending on how much each respondent wished to talk; the shortest took 35 min, the longest 90 min.

All interviewees (whether previously known to the researcher or not) were treated in the same way. The interviews started with an introduction of the researcher’s position, whose background was outside of H&SC, and who had no professional ‘allegiance’. No questions were excluded for any respondent. While knowing some of the participants could lead to a critique of ‘objectivity’, the research was conducted on the premise that ‘all accounts of the world are…constructed on the basis of particular assumptions and purposes’ (Hammersely and Gomm 1997, p. 5) and that as such, complete objectivity is unobtainable. From this perspective, the results of research would always be based upon a personal and unique interpretation of the data. The fact that some participants were known to the researcher is therefore acknowledged as important contextual information that is recognised and reflected upon where relevant in the analysis.

The foundation of the analysis was notes that were taken during each interview, with interviews transcribed verbatim from full audio recordings as soon as possible after occurrence, enabling what Rossman and Rallis (2003, p. 271) describe as ‘learning as you go’. Interview transcripts were annotated with immediate thoughts while transcribing, with previous transcriptions and notes re-visited before conducting further interviews. The coding was undertaken manually, through multiple readings of the data, and by writing notes and suggested code headings on to interview transcripts. This inductive approach was chosen as being the most appropriate for explorative inquiry, allowing for themes to emerge from the data, rather than fitting these into a pre-existing coding frame built upon the preconceptions of the researcher (Braun and Clarke 2006). The analysis itself was thematic, for both the narrative elements and open-ended responses to questions in the interviews. While thematic analysis is one of a number of recognised methodologies of analysing narrative data (Riessman 2008), this seemed the most appropriate way to deal with both the volume of data and the fact that interviews contained a mixture of narrative and descriptive responses. Codes were created for phrases, statements or narrative that recurred between interviews or specifically addressed one or more of the research questions (Koh et al. 2014). Sub-codes were created for minority opinions or for opinions that differed from the majority of opinions expressed. A number of interesting themes emerged; some directly related to the research questions, others of interest around the topics of professional identity and interprofessional education. The results presented here represent a small proportion of the final themes, but are those which have been identified as having the largest implication for those involved in H&SC education.

The research was granted ethical approval by the Leeds East Research Ethics Committee, the Leeds Teaching Hospitals NHS Trust R&D service and the University of Leeds Faculty of Medicine and Health Research Ethics Committee.

## Findings

33 staff were interviewed in total. The number of representatives from each profession is detailed in Table 1. As described in the methods, due to the recruitment method, some (9/17) of the academic staff were already known to the researcher, although none were close colleagues (i.e. that worked within the same team). None of the practicing staff were known to the researcher prior to the research; although all taught students on their wards, none were involved in further teaching activities for any HEI, and most had had very little or no involvement with any formal IPE projects.

**Table 1:** Current role or professional background of interview respondents

	Practicing staff	Academic staff
Audiologist	–	1
Dietician	2	1
Medic	4	2
Midwife	1^a^	1^a^
Nurse	2	5^a^
Occupational therapist	2	2
Pharmacist	2	–
Physiotherapist	1^a^	1
Radiographer	–	1
Speech and language therapist	2	–
Social worker	–	2
Vision impairment rehabilitation	–	1

^a^ Denotes that at least one respondent classified under a different professional role or background indicated that they also trained and worked for the indicated profession for a period of time

The focus of the findings presented here are those that relate to, or raise questions about, the concept of ‘interprofessional responsibility’ (IPR) which was an over-arching theme to emerge from all aspects of the research. The notion of IPR first presented itself during the literature review, when it appeared that much of what IPE was designed to achieve was about professional responsibilities towards colleagues and patients. As the interviews progressed, numerous elements of ‘responsibility’ appeared to be linked to the way in which individuals conceptualised their professional identities, and how this related to the way in which they worked with others to achieve the highest standards of patient care. Although the findings from the data are therefore being presented as something that contributed to the definition of IPR, its conceptualisation is being presented first, in order to allow for more nuanced discussion of the implications of the findings of the research as a whole for practice.

## Interprofessional responsibility

Interprofessional responsibility—(IPR)—is defined here as any instance where an individual professional or profession as a whole could be perceived as having a responsibility concerning interprofessional behaviour. ‘Responsibility’ itself is defined here as an accountability in terms of behaviours and thought processes, and includes the way in which professionals talk about their own identities; the way they talk about the professional identity of others, and the way in which staff act towards each other, in order to provide the highest standards of patient care. As a *concept,* it could be considered that IPR is not particularly new; certain levels of responsibility towards interprofessional behaviour are already suggested in the literature through proposals that H&SC students must be exposed to clinicians and educators who are interprofessional, and participate in authentic multi-disciplinary teams in clinical settings (Thistlethwaite 2012; Pollard 2008). However IPR describes more than this, encompassing both behaviours and identity. IPR builds on the suggestion that all professions incorporate ‘interprofessional socialisation’ (Khalili et al. 2013) into their training. In Khalili et al*.’s* model of interprofessional socialisation, it is proposed that students develop a ‘dual identity’ that incorporates interprofessional behaviours as part of the professional socialisation process. However, given that identity can be conceptualised as fluid and that individuals can be understood to have multiple, simultaneous identities (Lawler 2008), it is proposed here that IPR should be *part of* the professional identity of any member of any H&SC profession, not considered as something that is separate or distinct from one’s core professional identity. Thus IPR is proposed here as an improved way to encapsulate and articulate both the way in which professional identities are conceptualised, and describe behaviours concerning how professionals could and should be trained and practice.

As is apparent throughout the discussion of the findings, the notion of all professions incorporating such ‘responsibility’ into their identities raises questions about the extent to which professions—either individually or collectively depending on context—have a responsibility to engage with, share best practice concerning, or educate their own members about interprofessional education and collaborative practice, in order to provide the highest quality patient care. As the research here aimed to understand if interpretations of professional identity by members of H&SC staff have implications for the way IPE is developed and delivered, one question arising from this articulation of IPR is whether it helps to address any of these implications.

### Intra-professional identity

While most literature on the professional identities of H&SC staff discusses the identities of professions as a whole (see, for example, Hilton and Slotnick (2005) in relation to medicine, Hallam (2000) discussing nursing, Pollard’s (2011) description of midwifery, and Gibelmen’s (1999) review of ‘social work’, this did not relate to the way in which respondents identified themselves in this study. The majority of respondents, both academic and practicing, identified themselves as part of a branch, sub-unit or specialty of the profession in which they worked.

…a big part of the professional identity for me has been a learning disability nurse and I think that is quite unique within the family of nursing and also within the family of health and social care and I’m quite happy to describe myself as a learning disability nurse… I think that it brings with it um, a certain set of values and attitudes that I’d like other people to think I would have as a nurse and personally as well. (HEI03)

It depends who I’m talking to, well as a group we call ourselves specialist midwives so I suppose that’s how I think of myself. I still think of myself as a midwife but that I specialised in a slightly different role to most other midwives. (NHS09)

Indeed most respondents identified as having an ‘intra-professional identity’ rather than an identity described by their overarching profession. If this is generally true of H&SC professionals, there are implications for both the way in which both professional identity and IPE are conceptualised, with some respondents recognising this issue. A respondent working in child social work said he felt more ‘professionally aligned’ to both child- and learning disability nurses than to adult social workers; this sentiment was endorsed by nursing professionals aligning themselves to branches of social work. Similarly, occupational therapists (OTs) working in rehabilitation aligned their identities more closely to other rehabilitation professionals than to OTs working with children. It is likely that such perceptions are influenced by the way in which multi-disciplinary teams function, where professionals align closely to work together to achieve the same tasks.

For these respondents, an element of IPR already appears to be a key part of what they perceive their professional identity to be. Crucially, this responsibility towards working effectively with other professions was also associated with doing what is best for patients. The main questions arising here are how to establish most effectively such an identity and associated attitude in student H&SC professionals and how to understand whether this can be done via, or with the help of, IPE. Conversely, if students are not exposed to the notion of IPR early enough, it may be that their professional identity will suffer, as they could potentially struggle with the difference in expectation between what they feel their identity is supposed to be and the reality of a much more multi-disciplinary role, or even perceive they have a ‘weak’ identity in comparison to others. For students professionals in particular, it is vitally important that all contributions to teams as interprofessional H&SC professionals should be recognised and valued.

### Professional identity becomes ‘stronger’ with experience

Respondents did not often indicate that their profession had a strong professional identity (to some extent, participants suggested that admitting to this was implied as a negative trait), but many claimed that they personally had what they considered a ‘strong’ professional identity. This was often associated with a strong level of identification with what they saw as the core values of their chosen profession. However, it was also apparent that more senior staff were more likely to claim that their professional identity was strong. Staff who had more recently qualified reported that they were yet to develop a stable professional identity, something they ascribed to the rotational nature of their roles. Nevertheless, there was recognition among these participants that they would likely achieve a more stable identity once they held a more permanent role:

I think because I’m a junior member of staff, and as I said a rotational member of staff at the moment, my identity fluctuates and changes quite a lot – I suppose you are a bit of a chameleon at this stage in training…I wouldn’t say I have an allegiance or that this is where I see myself going as a specialist*…* (NHS12- doctor)

Talking generally about the concept of professional identity (rather than specifically about their own), other respondents amplified these thoughts concerning senior status and identity:

I think the more senior you get, the more people acknowledge your professional identity. (NHS09 - midwife)

I would imagine [that] as you get more senior and you have more influence that you would feel even more that you had some sort of stronger identity. (NHS13 – occupational therapist)

The idea that professional identity becomes more secure with experience is not surprising (indeed, many respondents acknowledged that their identity had changed over time and would continue to change), but it is important in the context of one other emergent theme: that collaborative practice is perceived to be learned most effectively through experience, which is discussed later in the findings.

### Negative stereotypes

Despite respondents’ awareness of the research topic, and all interview respondents saying they were fully ‘signed-up’ to the concepts of interprofessional education and improving collaborative practice (regardless of whether they had had any prior involvement in IPE or not), many expressed opinions concerning other professions that could have been perceived as ‘negative’:

The doctor is still king and if he isn’t then he still thinks he is. They’ve maybe come down a peg or two to a lower prince. (NHS3 – speech and language therapist)

I think physios just fix bones and muscles, don’t they? I think nurses have become confused, she says in an opinionated way. I think the role of nursing has really changed…and are they now pseudo-doctors? …social workers sort of know what they do. They’re just bank managers aren’t they? (HEI08 – occupational therapist)

You know there are different nuances with different professional groups, I always think that dieticians are quite picky; speech and language therapists are…very attention to detail type thing; I think physios have…a broader view of things generally, and OTs tend to be very particular. (HEI09 – dietician)

Examples of such ‘leaked’ opinions about other professionals were more common in interviews with academic staff than with NHS staff. One possible reason is that the researcher had previously worked with a number of the academic interviewees; it may have been the case that these respondents were more open and honest in their opinions. Alternatively, these responses may be more about who the respondents were and what they had experienced, reflecting views of staff who were not currently practicing. It is also possible that some HEI staff are less in touch with the reality of changing working practices; such a finding ties in with Frenk et al.’s (2010) example that there is a gap of about 20 years between the development of team-based practice in the workplace and the concept being introduced in educational settings. Nevertheless, examples of negative comments were evident across the whole range of interviews. Such comments lead to concerns for a number of reasons. That they exist at all suggests that it is difficult for some professionals not to characterise other professions (or even their own) without resorting to stereotypical and somewhat negative views. Additionally, given the influence of staff members on the socialisation processes of trainee professionals, the willingness to express stereotypically negative views of professions may mean that they are adopted and reinforced by students. Such findings also reinforce the relationship between identity and the proposed concept of IPR; and that one part of IPR that can be taught is the responsibility to refer to the role and identity of all professionals with respect.

### The theory and the reality of IPE in universities

All respondents were asked to describe their experiences (if any) of facilitating IPE. Only two of the NHS staff had any experience of doing so, and both suggested that they felt the sessions had positive impacts on students involved in the sessions. However, academic staff, all of whom were involved in IPE programmes to some degree, were more pragmatic concerning their institution’s approaches to the provision of IPE. Their responses were well illustrated by this quotation from one participant:

The first thing to understand is that things go in a circular – so we had IPW [interprofessional working] as a module, the evaluation would always then show that it would be better if it was threaded through the whole curriculum, so the next time we revalidate we spread it through the whole curriculum, somebody will come up with the bright idea that it would be better if you can consolidate it…I think the whole issue about being seen to do it rather than being trusted to do it drives us towards a more reductionist approach. (HEI04 - occupational therapist)

In addition to the ever-changing nature of IPE provision, the idea that benchmarks set by curriculum regulators drive policy on the provision of IPE meant that, for many academic respondents, the notion of ‘box ticking’ and being seen to be provide IPE was regarded as the most important factor in its provision. While this is not to suggest that effort does not go into providing IPE in the respondents’ institutions, there was a worrying undercurrent in academic responses which suggested that IPE provision was often more about paying ‘lip-service’ to it than focusing on changing working practices, either for students (future graduates) or even themselves. For example, respondents HEI14 and HEI15, both from the same institution, claimed that their profession (the same one) was excluded from interprofessional initiatives, because they had moved to a different building. The implication was that moving out of the same physical location as other H&SC professions meant it was no longer relevant for their students to have opportunities to work and learn from students of other professions. This discord between academic behaviours and the expectations of the clinical workplace is a further tension for students who may receive mixed messages, or worse, learn unwanted behaviours.

The recognition that IPE needs to be implemented in a way which is both timely and relevant for students did not always seem to fit with the descriptions about the way in which it is designed and implemented by educational institutions. The findings presented here have already highlighted that negative opinions of other professionals can and are expressed within educational environments. Similarly, the impact of negative experiences of IPE can be long-lasting on both opinions towards collaborative practice and subsequently on opinions of the other professions involved:

We did have IPL days at university – I think there was one a year. I hated them. We were put into groups with social workers, occupational therapists, speech and language therapists and dieticians, and we had to work through case studies. I was frustrated by someone in the group who was completely unprofessional, and it put me off the concept of IPE completely. (NHS 15 – dietician).

Although only a single case, it is interesting to note that this respondent was also very negative about her current job role, describing it as undefined and feeling that no other professionals really knew what she did. However it is proposed here that if IPR was incorporated into professional identity this would help to facilitate a change in attitude towards IPE provision, making it more meaningful and moving it away from the ‘box-ticking’ exercise, as well as ensuring that academics sought to better prepare students to take more from their work-based experiences.

### Effective collaborative practice is learned most effectively through experience

In line with some existing research (Jakobsen et al. 2011), many respondents expressed the view that effective collaborative practice could only be learned or become relevant through experience, and could not be ‘taught’ solely through IPE initiatives. This was essentially associated with difficulties involved in capturing the experience of effective collaborative practice and distilling this to students:

I think the challenge it to harness it – to capture it on a day-to-day basis. (HEI05 - nurse)

It’s not that they don’t get exposure, I just wonder whether we are not capitalising and using that and students need a lot of help signposting, that kind of thing. (HEI02 - midwife)

We could teach students for years and years and years, what we can’t give them in the classroom is experience. (HEI15 – social worker)

It comes through experience…knowing where to ask for help. (NHS06 - physiotherapist)

For other respondents, this was expressed through the recognition that effective team working often arises in better-established teams:

It’s about being able to put a face to a name – that breaks down barriers…Familiarity and trust come as a gradual thing. (NHS09 - midwife)

Nurses pick up on how different doctors conduct their ward rounds, but it’s informal and learned through observation. (NHS11 - doctor)

As learning by experience is impossible to teach in a classroom; such understandings of collaborative practice pose challenges for the conceptualisation of IPE as a system that can be ‘separated’ from other experiences and hence ‘taught’. This raises questions about whether introducing the concept of IPR into H&SC training and therefore into the core identities of all H&SC professions (including an awareness of the need to work well with other professions and learn to collaborate with them), would result in students who are better prepared to learn from placement-based experiences. The challenge for educators therefore becomes how they identify these potential learning experiences in practice and how they prepare students to learn from these. In particular, as IPR is by definition learned through experience, this should be something that is considered with more senior students who have the ability to see and practice IPR more legitimately, the message of which could be extended into early postgraduate training.

It could also be proposed that the fact that collaborative practice is often viewed as developing with experience is related to the earlier finding; that professional identity is perceived to become stronger with experience. From combining these two findings, it appears that as individuals develop stronger professional identities—or a sense of a professional identity—they are better able to work with other professions. This is not to suggest that one of these occurrences (a stronger professional identity or ability to work collaboratively) causes another, but that as both are perceived to develop with experience, it suggests that a stronger professional identity is *related* to the ability to collaborate across professional boundaries.

## Discussion: implications for practice

There is no single, homogenous experience of professional identity formation; all individuals have unique perceptions of their own experiences that contribute to how they feel about their identities. Nevertheless, the research conducted in this study identified a number of common themes concerning perception of identities that the research participants appeared to share, some of which may lead to further understandings of how perceptions of professional identity can impact upon both IPE and collaborative practice.

Firstly, it was apparent from interviews that existing academic interpretations and descriptions of ‘professional identities’ do not align with the way in which many H&SC professionals actually perceive their identity. In particular, there was a tendency for respondents to align their own identities with that of a branch or sub-group of a profession; this is defined here as an ‘intra-professional identity’. This suggests a need for further research in this area in order to understand the implications for IPE and collaborative practice. For example, in exploring whether if H&SC professionals are more likely to identify with an ‘intra-professional’ identity, does ‘IPE’ become a necessary mediator between branches of professions? Given the importance of relevance to the success of IPE (previously identified by Anderson and Thorpe 2008; Rosenfeld et al. 2011) it can be suggested that IPE should focus on ensuring that specific branches of professions have opportunities to learn and work together, leading to students having learning experiences that are relevant to their future working practices. Notably, some respondents indicated that their identity was already associated with working well with other professions to provide the best possible patient care; describing the fact that for some, IPR is already incorporated into their professional identity. The question that arises here becomes how such IPR can be incorporated into the professional identities of all trainee professionals.

There was recognition from participants in this study that certain aspects of professional life, including collaborative working practices and (in some instances) a stronger sense of professional identity, cannot be ‘taught’ and are rather learned only from experience (i.e. from practice). Instead of trying to ‘teach’ these things, this paper proposes that it is the responsibility of educators (and indeed inherent in their own IPR) to identify ways of preparing students to, first, understand that these skills will develop over time, and second, make the best use of placement experiences in order to learn how to strengthen them.

Educators on both an individual and organizational level have further interprofessional responsibilities in terms of the formal IPE that is offered; acknowledgements made in this study imply that at least some taught IPE is ‘tick-box’ rather than focused around best-practice recommendations; this has potential consequences for the way in which students view both IPE and collaborative practices. The need for students to be exposed to interprofessional experiences within authentic settings is thus paramount to both the understanding, and development, of IPR.

Finally, this study has identified that H&SC professionals have responsibilities in terms of the way in which they talk about all professions (including their own), and must remain mindful of how their spoken views can have influence on perceptions of identities and the socialisation of students. Expressing negative opinions of professions based upon unhelpful stereotypes, be they of one’s own profession or another, are likely to impact student opinion and perpetuate negative attitudes. In order to break this cycle of behaviour, students need to be trained to both recognise and deal with such opinions, given that fixed stereotypes and biases from senior professionals are unlikely to be changed, which risks such behaviour continuing.

It should also be recognised that with reference to both ‘box-ticking’, and the willingness to express negative opinions of other professions, there was an apparent gap in attitudes between academic and practicing staff. This is concerning, and while gaps in best practice are already acknowledged (Frenk et al. 2010), suggests that there is more research work, and more progress, to be made here.

Finally it is acknowledged that the development of identities, of IPE programmes and of collaborative practice do not take place in a vacuum. The influence of uncertain work environments and organisational systems, the influence of politics on healthcare systems, and competing curriculum drivers from professional bodies and higher educational institutions raise huge challenges for all those investing in endeavours to improve healthcare education in order to raise the standards of patient care. However, if the concept of IR could move the debates around professional identities away from thinking about differences and more towards the common aims held by each profession, then there should be value in developing the concept further and introducing it to both students, and of course, to staff.

## Limitations of the study

This is a one-off exploratory study conducted in one part of the UK. The analysis is the researchers’ own and as such the results presented here are one interpretation made from one persons’ reading of the data. Additionally, as identity is such a personal and situated concept, it is difficult to make any claims to generalisability, but the identifiable themes in respondents’ experiences of their own identity point to ways in which educators in particular can start to frame IPR as part of all professional identities. Recruitment for the study was not ideal, insofar as the researcher did know just over half of the academic interviewees. This familiarity may have influenced participants’ willingness to share, but in the emerging data it appeared that this resulted in more honest sharing of views. Recruitment for all the interviews was self-selecting volunteers, this could of course explain why all respondents were supportive of IPE and what it sets out to achieve if they were interested in taking part in a research project about it in the first place, this may have therefore influenced some of the responses given.

## Conclusions

Failures of patient care in the H&SC system are to some extent still attributed to the inabilities of H&SC professionals to communicate or work effectively together (Laming 2009; Francis 2013). Such findings highlight a need for continual focus on improving understandings of this element of H&SC work, and for further discussion of the relationship between professional identities, roles, boundaries and collaborative practice. The concept of ‘interprofessional responsibility’ has been introduced here and is proposed as something that would help all H&SC professions to reconceptualise their identity. All professionals have a responsibility to work across professional boundaries in order to ensure the best patient care is provided; they also need to understand these responsibilities in the context of their job roles. It is proposed that the concept of IPR can open up debate around how this is best achieved together, and work as a concept that can be introduced early on to student professionals and built on over time. In this way, IPR could be introduced as a core element of each individual professions’ identity. It is also proposed that the incorporation of IPR would change professional practices, helping students understand from the outset of their professional careers the responsibilities towards working together effectively that will ultimately enable them to provide the best possible care to all patients and service users.

## Appendix: interview guide

- Ask respondent to describe their professional background and their current job role.
- First of all I’d like to ask you about some of your own experiences of interprofessional education and working. Was there an emphasis on interprofessional education and working as part of your own professional training? [Explore—what, classroom or practice based, which other professions involved, undergraduate or postgraduate]
- What sort of interprofessional working do you and your staff engage in now? [Explore—context—is this every day? How easy or difficult is it to define interprofessional working compared to ‘non-interprofessional’ working?]
- And do you work or supervise students in practice? [If yes] Are students introduced to interprofessional team working when they come to work in practice? [explore—what knowledge of interprofessional working do students tend to come to their placements with]
- Interprofessional education aims to improve the communication and team-working skills of those who undertake it—which has the ultimate aim of improving patient (or service user) care. How successful do you think interprofessional education—or working—is in achieving this aim?
- Are there some professions which you find it easier to work with than others? [Explore—is there an organizational structure which means you end up working with some professions more than others?]
- A slight change of topic now—thinking a bit more about the idea of ‘professional identity’. Do you feel you have a strong ‘professional identity’? [Explore—do you describe yourself by your profession in and out of work? Is the concept of ‘professional identity’ outdated?]
- Do you think some health and social care professions have a stronger professional ‘identity’ than others?
- Do you think professional identity is something that you develop? [Explore—do you think people start their training with a fixed idea of what their ‘professional identity’ will be?]
- I’d like now to talk a little bit about the work of the ALPS CETL and its programme of interprofessional work and assessment. Can you just briefly outline for me how, if at all, you are involved with ALPS? [Explore—how became involved, how ALPS was introduced in practice]
- And are you aware of, or have you been involved in, any other large scale interprofessional education initiatives? [Explore—scale of initiatives, how involved].
- Do you think that ALPS—and similar programmes of work—have, or will—make a difference to the way in which health professionals think about interprofessional education and working?
- And do you think that an increased amount of focus on interprofessional education will change the way that people think about their professional identity? [Explore—impact on perceptions of health professionals versus perceptions of public/patients/service users].
- Finally, one of the main aims of ALPS is to introduce a series of generic skills assessments to undergraduates. So for skills such as communication, team-working and ethical practice, the aim is that any student could be assessed by any qualified member of another health and social care profession. I’d be interested to know what you think the benefits and challenges of introducing this interprofessional assessment for generic skills are.
